# Educational intervention on perceived stress among adults with type 2 diabetes and metabolic syndrome: a non-randomized clinical trial

**DOI:** 10.17533/udea.iee.v42n1e03

**Published:** 2024-04-27

**Authors:** Wilkslam Alves de Araújo, Isleide Santana Cardoso Santos, Randson Souza Rosa, Diego Pires Cruz, Cícero Santos Souza, Rita Narriman Silva de Oliveira Boery, Claudia Geovana da Silva Pires, Andréa dos Santos Souza, Roseanne Montargil Rocha

**Affiliations:** 2 RN, Ph.D. Professora. Email: isantana@uesb.edu.br Universidade Estadual do Sudoeste da Bahia Brazil isantana@uesb.edu.br; 3 RN, M.Sc. Email: enfrandson@gmail.com Universidade Estadual de Feira de Santana Brazil enfrandson@gmail.com; 4 RN, Ph.D. Email: diego_pcruz@hotmail.com Universidade Estadual do Sudoeste da Bahia Brazil diego_pcruz@hotmail.com; 5 MD, Email: cicerossz@hotmail.com Universidade Estadual do Sudoeste da Bahia Brazil cicerossz@hotmail.com; 6 RN, Ph.D. Email: rboery@gmail.com Universidade Estadual do Sudoeste da Bahia Brazil rboery@gmail.com; 7 RN, Ph.D. Email: cgspires@ufba.br Universidade Federal da Bahia Brazil cgspires@ufba.br; 8 RN, Ph.D. Email: assouza@uesc.br Universidade Estadual do Sudoeste da Bahia Brazil assouza@uesc.br; 9 RN, Ph.D. Professora. Email: rmrocha@uesc.br Universidade Estadual do Sudoeste da Bahia Brazil rmrocha@uesc.br; 10 Graduate Program in Nursing and Health, Universidade Estadual do Sudoeste da Bahia, Bahia-Brazil. Universidade Estadual do Sudoeste da Bahia Graduate Program in Nursing and Health Universidade Estadual do Sudoeste da Bahia Bahia Brazil; 11 Graduate Program in Collective Health, Universidade Estadual de Feira de Santana, Bahia- Brazil. Universidade Estadual de Feira de Santana Graduate Program in Collective Health Universidade Estadual de Feira de Santana Bahia Brazil; 12 Graduate Program in Nursing and Health, Universidade Federal da Bahia, Bahia-Brazil. Universidade Federal da Bahia Graduate Program in Nursing and Health Universidade Federal da Bahia Bahia Brazil

**Keywords:** diabetes mellitus, type 2, community health nursing, stress, psychological, health promotion, metabolic syndrome., diabetes mellitus tipo 2, enfermería en salud comunitaria, estrés psicológico, promoción de la salud, síndrome metabólico., diabetes mellitus tipo 2, enfermagem em saúde comunitária, estresse psicológico, promoção da saúde, síndrome metabólica.

## Abstract

**Objective.:**

To assess the effectiveness of an educational intervention on perceived stress and metabolic syndrome parameters among adults with type 2 diabetes mellitus.

**Method.:**

Fifty-one adults (aged 48.73±7.84; 86.3% of women) were included in a non-randomized clinical trial performed in a healthcare unit for six months (Brazilian Clinical Trial Registry: RBR-43K52N). All participants were diagnosed with type 2 diabetes mellitus and metabolic syndrome (intervention group, n=26; control group, n=25). The intervention consisted of a nurse-led educational health-promoting program with a multidisciplinary approach organized in seven workshops. The primary outcome was decreased perceived stress, and the secondary outcome was improvement in metabolic syndrome parameters according to perceived stress levels. These outcomes were assessed at two points in time, at the baseline and follow-up.

**Results.:**

Participation in the intervention program resulted in a significant decrease in perceived stress (p=0.028). The stressed participants in the intervention group experienced a significant decrease in blood glucose levels (p=0.001) and a significant increase in high-density lipoprotein-cholesterol (p=0.003) concentrations after the six-month intervention.

**Conclusion.:**

The nurse-led educational health-promoting program decreased perceived stress among adults with type 2 diabetes mellitus and metabolic syndrome, improving fasting blood glucose and high-density lipoprotein cholesterol among the stressed participants in the intervention group.

## Resumen

**Objetivo.:**

Evaluar la efectividad de una intervención educativa sobre el estrés percibido y los componentes del síndrome metabólico en adultos con diabetes mellitus tipo 2.

**Métodos.:**

Se incluyeron 51 adultos (48.73±7.84 años; 86.3% mujeres) de un estudio no-ensayo aleatorizado realizado en una unidad de salud durante seis meses, con Registro Brasileño de Ensayos Clínicos: RBR-43K52N, todos los participantes fueron diagnosticados con diabetes mellitus tipo 2 y síndrome metabólico (grupo intervención, *n*=26; grupo control, *n*=25). La intervención consistió en un programa educativo de promoción de la salud con enfoque multidisciplinario, liderado por una enfermera, estructurado en siete talleres grupales. El resultado primario fue la reducción del estrés percibido y el secundario, la mejora de los componentes del síndrome metabólico influenciados por el nivel de estrés percibido, evaluado en dos momentos, al inicio y después del seguimiento.

**Resultados.:**

La participación en el programa de intervención resultó en una reducción significativa del estrés percibido en comparación con el grupo control (*p*=0.028). Los participantes estresados ​​en el grupo de intervención tuvieron, respectivamente, una disminución y un aumento significativos en las concentraciones séricas de glucosa (*p*=0.001) y lipoproteínas de alta densidad-colesterol (*p*=0.003) después de seis meses de intervención.

**Conclusión.:**

Un programa educativo de promoción de la salud liderado por enfermeras fue eficiente para reducir el estrés percibido entre adultos con diabetes mellitus tipo 2 y síndrome metabólico, además de mejorar la glucemia en ayunas y el colesterol unido a lipoproteínas de alta densidad en los participantes del grupo estresado de intervención.

## Resumo

**Objetivo.:**

Avaliar a efetividade de uma intervenção educativa sobre o estresse percebido e os componentes da síndrome metabólica em adultos com diabetes mellitus tipo 2.

**Métodos.:**

Foram incluídos 51 adultos (48.73±7.84 anos de idade; 86.3% mulheres) em um ensaio clínico não-randomizado realizado em uma unidade de saúde durante seis meses, com Registro de Ensaio Clínico Brasileiro: RBR-43K52N.Todos os participantes apresentavam diagnóstico de diabetes mellitus tipo 2 e síndrome metabólica (grupo intervenção, *n*=26; grupo controle, *n*=25). A intervenção consistiu em um programa educativo de promoção da saúde com abordagem multidisciplinar, liderado por enfermeiro, estruturado em sete oficinas em grupo. O desfecho primário foi a redução do estresse percebido, e o secundário, a melhora dos componentes da síndrome metabólica conforme influência do nível de estresse percebido, avaliados em dois momentos, na condição basal e após o acompanhamento.

**Resultados.:**

A participação no programa de intervenção resultou na redução significativa do estresse percebido em comparação com o grupo controle (*p*=0.028). Os participantes estressados do grupo intervenção tiveram, respectivamente, diminuição e aumento significativos das concentrações séricas de glicose (*p*=0.001) e da lipoproteína-colesterol de alta densidade (*p*=0.003) após seis meses de intervenção.

**Conclusão.:**

Um programa educativo de promoção da saúde liderado por enfermeiros foi eficiente para reduzir estresse percebido entre adultos com diabetes mellitus tipo 2 e síndrome metabólica, além de causar melhora da glicemia de jejum e e da lipoproteína-colesterol de alta densidade dos participantes estressados do grupo intervenção.

## Introduction

The prevalence of metabolic syndrome (MS) has increased at an alarming rate, a silent epidemic considered a global public health problem. MS has often been associated with type 2 diabetes mellitus (DM2), which is also one of the most significant global emergencies. However, the determinants of both conditions are modifiable, e.g., perceived stress.^1^ Perceived stress concerns the degree to which an individual perceives life experiences as stressful.^1^ The imbalance produced between efforts demand and low reward in a multimorbidity context, i.e., in the presence of ≥ 2 chronic conditions, especially DM2 and MS, is considered a significant risk factor for an individual’s mental health, as it results in increased psychological stress. Studies show that middle-aged individuals experiencing high-stress levels are at a greater risk of presenting MS than their counterparts experiencing low-stress levels.^2^^,^^3^ A meta-analysis shows a significant association between perceived stress and abdominal obesity and lipid parameters that define MS.^4^ In any case, the mechanisms behind the relationship between perceived stress and MS seem to be related to an increase in body obesity; however, such an explanation is complex and require further investigation. One of the current explanations concerns individuals’ low ability to adapt to chronic stress mediated by neuroendocrine disorders, mainly due to changes in the hypothalamic-pituitary-adrenal axis, which causes an increase in catecholamines and serum cortisol levels, which, in turn, leads to a decreased appetite control and increased adiposity. As a result, individuals experience increased blood pressure, higher glucose levels, and the accumulation of circulating lipids.^5^

This syndrome comprises a set of metabolic disorders with cardiovascular risks, such as central fat deposition and insulin resistance (IR), and is associated with mortality in adults with DM2.^1^ Furthermore, the number of adult individuals with DM2 and MS considerably impacts the country’s health status and overall healthcare costs. Therefore, applying knowledge and skills from the different fields of knowledge, including nursing, in the planning and implementation of health-promoting programs to deal with the increased burden of chronic diseases associated with perceived stress is urgent and necessary to effectively implement health policies directed to this population and ensure adequate monitoring. It is advisable to decrease the impact of MS by reducing its incidence in the primary health care (PHC) scope, as this is the healthcare level where individuals with a high cardiometabolic risk, such as those unable to deal with stress, concentrate.^6^ The chronic nature of DM2 and MS demands lifestyle changes. Hence, self-care should be encouraged, and strategies to cope with stress should be promoted among individuals within the PHC. Nonetheless, these actions remain a challenge, especially when there is also an intention to relieve the burden of psychological stress. Adopting a healthy lifestyle is essential in preventing such conditions’ alarming rates. Recognizing such complexity and adopting a healthy lifestyle is essential to prevent these diseases. In this context, nurses play a vital role in implementing policies and ensuring adequate monitoring and promoting the health of this vulnerable population.^1^

Although incipient, recent advancements concerning perceived stress and MS enable the investigation of these relationships. The Perceived Stress Scale (PSS) is one of the most popular subjective tools for assessing psychological stress, though only a few studies have addressed the association between perceived stress and diabetes or MS.^6^^,^^8^^,^^9^Therefore, this study aimed to assess the effectiveness of an educational intervention on perceived stress and MS parameters among adults with DM2.

## Method

A two-arm non-randomized clinical trial was performed considering a six-month educational intervention registered in the Brazilian Clinical Trial Registry (REBEC) under code RBR-43K52N. This study is part of the research project “*Cuidar educando na síndrome metabólica*” [Providing care by teaching metabolic syndrome” (Opinion Report No. 2,850,239), conducted in a PHC unit in the urban area of Jequié, BA, Brazil.

The research team invited men and women between 18 and 59 to participate in the study during their usual appointments to care for high blood pressure and DM2 at the health unit. This first contact followed a standardized screening protocol to assess volunteer eligibility. All the participants had a diagnosis of DM2 and also met the criteria for MS, which requires at least three of the following: (1) waist circumference >102 cm for men and >88 cm for women; (2) triglycerides ≥150 mg/d; (3) HDL-c <40 mg/dl in men and <50 mg/dl in women; (4) blood pressure ≥130/85 mmHg; and (5) fasting blood glucose ≥100 mg/dl.^10^ Exclusion criteria were getting pregnant or missing >50% of the workshops.

**Figure 1:**
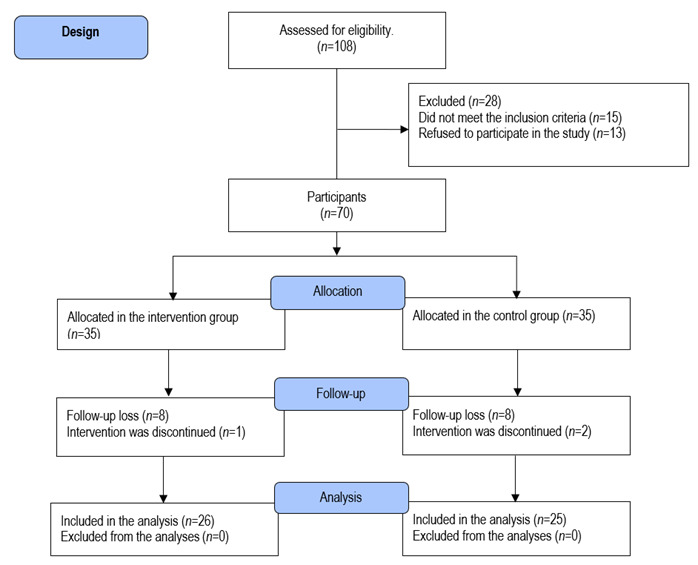
Flowchart. Participants’ distribution

Of the 108 adults selected for the eligibility analysis, 38 did not meet the inclusion criteria; hence, 70 adults (68.4%) with diabetes and MS remained and were intentionally allocated to the intervention or control group (35 individuals in each group). However, nine participants were excluded from the intervention group: one because she became pregnant and another eight due to low attendance at the workshops. Ten individuals were excluded from the control group because two moved to another city, and eight were not interested in continuing the study. Therefore, 51 individuals (26 in the intervention group and 25 in the control group) completed the educational intervention protocol and were included in the analyses. Figure 1 shows the participants’ distribution.

First, all participants in the intervention and control groups received general information about MS and individual information regarding how many MS criteria they met and their respective metabolic risks. The educational program was developed with Pedagogical Autonomy^11^ to promote the health of the participants in the intervention group. The program was led by nurses and organized into seven monthly workshops lasting from 90 to 120 minutes each. The workshops were held in groups at the health center after routine care was provided. Additionally, the workshops were organized in two parts: the first part included the participants being welcomed and the nurses addressing the content based on clinical guidelines for adults with MS^12^^-^^14^. The second part included a guest (health professionals) who talked to the participants about the group’s topics of interest, which were chosen at the end of each workshop. The topics covered in the program were MS aspects and risk factors (i.e., concept, diagnosis, treatment, complications, and behavioral changes), healthy diet, sedentary lifestyle, pain, stress and anxiety, ergonomics, integrative activities, spirituality, and metabolic and cardiovascular disorders. The multidisciplinary approach included the participation of the following guests: a physical educator, a nurse, a physical therapist, a pharmacist, a cardiologist, a nutritionist, and a psychologist. The nurses who conducted the program are researchers and members of the Health and Quality of Life Research Group at the *Universidade Estadual do Sudoeste da Bahia (UESB)* and received identical instructions and training to implement the intervention.

The control group did not participate in the educational program but, like the intervention group, continued receiving the usual care at the health center with monthly consultations. The participants in the control group also received a monthly telephone call to confirm their participation in the study and attended the health center according to their appointments. Apart from the scheduled measurements, there was no additional contact between the researchers and the control group during the study period.

The Institutional Review Board at UESB approved all procedures, and the participants signed free and informed consent forms after receiving detailed clarification. The participants were assessed at two points: before the intervention and at the follow-up (six months after the intervention). Data that characterized the sample were collected at baseline through individual interviews using a structured sociodemographic questionnaire (i.e., age, sex, self-reported race, years of schooling, and general health aspects, e.g., duration of diabetes). MS was determined via the NCEP-ATP III criteria. Abdominal circumference was measured horizontally at the midpoint between the iliac crest and the lower costal margin, using a flexible and inelastic measuring tape with 0.1 cm accuracy. Weight was measured with barefoot individuals dressed in light clothing on a portable digital scale (Wiso®, model W801) with a 0-180 kg capacity and 0.1 kg accuracy. Height was measured using a portable metallic stadiometer (Sanny, capriche model) with a 0.1mm resolution. Body mass index (BMI) was calculated with the participant’s weight in kilograms divided by the square of their height in meters.^15^ Blood pressure was measured with a validated semi-automatic device^16^ (Omron, model HEM-742 INT) and met the criteria by the Brazilian Hypertension Guidelines.^17^ The average of two readings of systolic and diastolic pressure measurements was used. Blood samples were taken from the antecubital vein, after 12 hours of fasting was confirmed, in a collection room prepared at the health center. Concentrations of serum triglycerides, HDL-c, and fasting blood glucose were measured by enzymatic methods (Roche Diagnostics).

The PSS, developed by Cohen, Karmarck, and Mermelstein in 1983 and later translated and validated by Luft et al.^18^, was used to assess stress. It is a 14-item scale, with seven positive and seven negative items. The negative items measure the respondents’ lack of control and negative affective reactions, while the positive items measure the ability to cope with stress. Each item is rated on a five-point scale, ranging from 0 (never) to 4 (always). Its final score ranges from 0 to 56 and represents an individual’s perception of stress over the last 30 days; higher scores indicate higher stress levels, and lower scores indicate lower levels of perceived stress. In this study, the total PSS scores were classified under two distinct categories, with a cutoff point ≥28 for the “stressed” category and <28 for the “non-stressed” category; this cutoff point was based on a similar study.^19^

We used the larger project database from March 15^th^, 2020. The sample calculation showed that 80 participants would provide a statistical power of 80%, considering an effect size of 0.25, an alpha error of 5%, and a sample loss of 20%. Mean, standard deviation, frequency, and percentage were used to report data. Normal distribution was verified using the Shapiro-Wilk test, and Levene’s test was used to analyze the homogeneity of variance. The Student’s t-test and the Chi-square or Fisher’s Exact test were used to compare the variables between the two groups (intervention and control) at the baseline. All comparisons between the groups were based on intention-to-treat analysis using multiple imputation. Two-way (time*group) ANOVA for repeated measures was used to assess changes in perceived stress and its influence on MS parameters from the baseline to the six-month follow-up considering all participants; F and p values were reported. Bonferroni post hoc was adopted to identify differences between pairs. All statistical analyses were performed using SPSS (version 24.0); significance was set at p<0.05.

## Results

The participants’ characteristics are presented in Table 1. Data from the 51 participants who completed the study was verified to analyze the effectiveness of the intervention program. No significant differences were found between the groups at baseline for any sample characteristics. The participants were aged 48.73±7.84 on average. Most were women (86.3%), non-Caucasians (78.4%), and had a low educational level (52.9%), with a DM2 duration from 1 to 10 years (60.8%). Based on BMI (32.33±6.08 kg/m2), the participants were overweight (27.5%), or presented class I (29.4%) or class II obesity (27.5%). The average perceived stress score was 25.55±8.89 at the baseline, i.e., most individuals were not stressed (56.9%). The mean MS diagnostic criteria score was 4.05±0.75, and according to the NCEP ATP III definitions, all the MS parameters were altered. 

**Table 1:** Participants’ characteristics at the baseline

Characteristics	Total (*n*=51)	**Intervention (*n*=26)**	**Control (*n*=25)**	p-value
Age (years), mean ± SD	48.73±7.84	48.96±8.03	48.48±7.80	0.829
Sex, n (%)				
Male	7 (13.7)	5 (19.2)	2 (8.0)	0.419
Female	44 (86.3)	21 (80.8)	23 (92.0)	
Race, n (%)				
Caucasian	11 (21.6)	4 (15.4)	7 (28.0)	0.324
Non-Caucasoam	40 (78.4)	22 (84.6)	18 (72.0)	
Years of schooling, n (%)				
< 8 years of schooling	27 (52.9)	15 (57.7)	12 (48.0)	0.488
≥ 8 years of schooling	24 (47.1)	11 (42.3)	13 (52.0)	
Diabetes duration				
< 1 year	9 (17.6)	3 (11.5)	6 (24.0)	0.184
1 to 10 years	31 (60.8)	19 (73.1)	12 (48.0)	
≥ 10 years	11 (21.6)	4 (15.4)	7 (28.0)	
Anthropometrics, mean ± SD				
Height_(cm)_	156.75±0.81	157.77±0.07	155.68±0.88	0.363
Weight_(kg)_	79.50±16.76	79.43±12.90	79.57±20.29	0.976
BMI_(kg/m2)_	32.33±6.08	31.86±4.38	32.81±7.53	0.582
Perceived Stress				
Score, mean ± SD	25.55±8.89	26.54±9.50	24.52±8.27	0.423
Stressed, n (%)	22 (43.1)	13 (50.0)	9 (36.0)	0.313
Not stressed, n (%)	29 (56.9)	13 (50.0)	16 (64.0)	
Metabolic syndrome				
Waist circumference _(cm)_	105.69±12.73	107.23±9.24	104.08±15.60	0.382
Triglycerides_(mg/dL)_	165.80±27.70	169.88±29.30	161.56±25.84	0.288
HDL-c_(mg/dL)_	42.02±10.54	39.42±9.38	44.72±11.18	0.074
Systolic blood pressure_(mmHg)_	139.06±17.95	140.04±16.73	138.04±19.43	0.695
Diastolic blood pressure_(mmHg)_	85.33±10.78	85.08±10.62	85.60±11.16	0.865
Glucose_(mg/dL)_	174.62±38.97	180.23±38.99	168.80±38.87	0.300
MS Score, mean±SD	4.05±0.75	4.15±0.83	3.96±0.67	0.367

Student’s t-test, Chi-square of Fisher’s Exact test. BMI: body mass index, HDL-c: high-density lipoproteins-cholesterol, MS: Metabolic syndrome.

**Figure 2:**
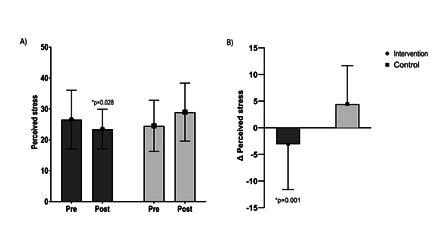
Comparison of perceived stress between the control and intervention groups at the pre- and post-interventions, Anova *Two-Way*

Figure 2 compares perceived stress scores at the pre- and post-intervention. The health-promoting educational program was found to significantly decrease the mean of perceived stress score among the intervention participants compared to the control participants (26.54±9.50-23.46±6.43 intervention group*vs.*24.52±08.27-28.96±9.41 control group, p=0.028; Figure A). The mean variation in stress levels was also significantly lower among the intervention participants (Δ= -3.07±8.51 intervention group*vs.*4.44±7.22 control group, p=0.001; Figure 2B).

**Figure 3:**
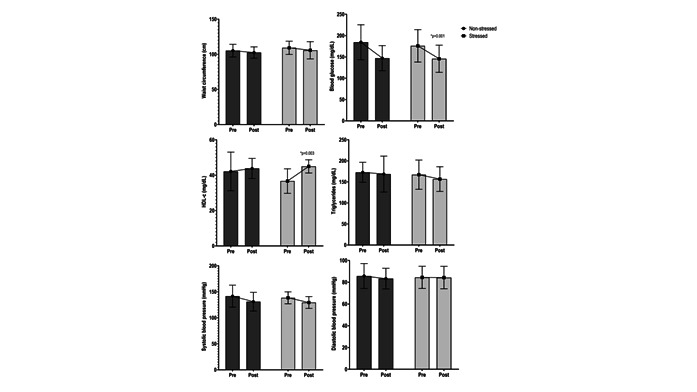
Clinical behavior of the intervention’s effectiveness on MS parameters according to perceived stress in the intervention group (non-stressed individuals, n=13; stressed individuals, n=13), Two-Way Anova

**Figure 4:**
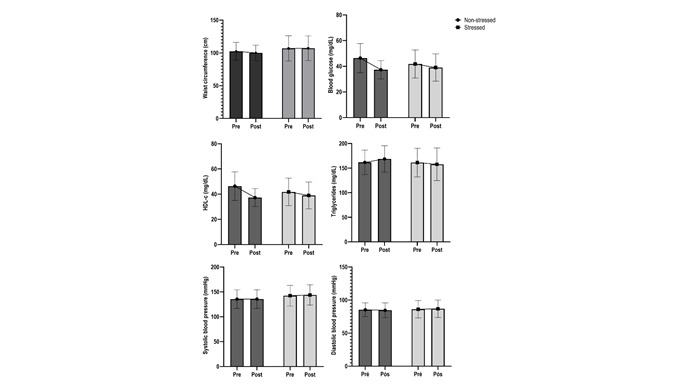
Clinical behavior of the intervention’s effectiveness on MS parameters according to perceived stress in the control group (non-stressed individuals, n=16; stressed individuals, n=9), Two-Way Anova

Figures 3 and 4 show that the individuals in each group were stratified according to the classification adopted for perceived stress (scores ≥28 indicate stressed individuals, and <28 indicate non-stressed individuals). The objective was to analyze the influence of perceived stress on MS parameters. The analysis showed a significant interaction for blood glucose (p=0.001) and HDL-c (p=0.003). A significant difference in blood glucose was found between stressed individuals in the intervention group (-31.59 mg/dl, 176.00±38.05 - 144.41±32.89 mg/dL) and non-stressed individuals in the control group (+35.56 mg/dl, 171.81 ±40.51 - 207.37±36.85) (p=0.026). Stressed individuals in the intervention group experienced a more significant increase in HDL-c concentrations (+6.46 mg/dl, 171.81±40.51-207.37±36.85) than those classified in the other categories (1.6 mg/dl, 42.15±10.91*vs.*43.71±5.49 NS-Intervention; -2.77 mg/dl, 41.77±10.92*vs.*39.00±10.63 S-Control; -9.06 mg/dl, 46.37±11.33*vs.*37.31±7.11 NS- Control).

A tendency toward a decreased mean of MS criteria was found in the intervention group, regardless of the stress level. However, stressed participants who received the intervention showed a higher decreased mean variation than those in the other categories (Δ=-0.69±1.03 S-Intervention; 0.33±0.70 S-Control; -0.38±1.26 NS-Intervention; 0.06±0.77 NS-Control), though without statistical significance (p=0.068).

## Discussion

This study showed that a nurses-led educational health-promoting program with a multidisciplinary approach significantly decreased perceived stress among adults with DM2 and MS six months after the intervention. Furthermore, the relationship between perceived stress and MS parameters was verified, with the results indicating a significant interaction with fasting blood glucose and HDL-c. Hence, the intervention group participants with high-perceived stress were more likely to experience decreased blood glucose levels and increased HDL-c concentrations. Additionally, the average number of MS criteria decreased in the intervention group regardless of the stress level, though without statistical significance. These findings imply that encouraging a healthier lifestyle may improve perceived stress and the number of MS criteria over time. The differences in stress levels among the participants may influence changes in the MS parameters; however, further investigation is needed. All the participants who completed the study showed an interest in continuing to attend the meetings. Such an interest is very relevant because there is a need for long-term health-promoting programs addressing MS in PHC units; the most common reasons for dropout included follow-up loss and poor adherence.

The baseline characteristics of the individuals who remained in the project to care for their health, focusing on MS, align with previous studies.^2^^,^^6^^,^^9^ In general, obese women with a low educational level and an average score of 25.55±8.89 on the perceived stress scale predominated. The frequency of obesity and MS was statistically significant among stressed individuals. However, the mean score of individuals with DM2 and MS on perceived stress was higher than in other populations.^2^^,^^5^A cohort study shows a prevalence of perceived stress of 10.13% in adults with cardiovascular risk factors, including MS (aged 54.2±9; 62.7% of women). An independent association was also found between perceived stress and obesity among men and carotid atherosclerosis among women. Additionally, individuals with chronic stress were significantly more sedentary (56.3%) and obese (48.4%) than their counterparts without chronic stress.^5^

Although the PSS scale has been used in various populations, few studies assess perceived stress using this scale in adults with DM2 and MS in the context of primary health care.^6^^,^^9^ Therefore, comparisons are limited. On the other hand, a program led by Morga et al.^9^ with regular exercise combined with psychoeducation, though without nurses leading the intervention, shows a decrease in the level of stress among older women with MS (aged 69.35±7.20). Another program reported data similar to our study, with a decrease in the perceived stress level of older women (aged 68.6±6.5) three months after an intervention including physical exercise, dance, health promotion education, and health psychoeducation administered in groups.^6^ One of our studies shows a mean score on perceived stress of 27.73±9.17 among middle-aged women (aged 47.69±8.15) with MS: 49.3% experienced stress, and 70.7% had DM2. Furthermore, stressed women obtained higher scores on perceived stress and presented lower HDL-c concentrations than non-stressed women.^20^ Perceived stress has been linked to changes in the lipid parameters of MS, especially HDL-c, which is consistent with our results.

According to the literature, high levels of perceived stress considerably increase the chances of MS prevalence.^2^^,^^8^Additionally, perceived stress is strongly correlated with cortisol levels. When associated with catecholamines, high cortisol levels appear to reduce appetite control, increasing fat accumulation and causing an increase in glucose serum levels. In a chronic state, it induces insulin resistance and lipid accumulation;^20^^-^^21^ high levels of circulating cortisol contribute to MS. Therefore, decreased blood glucose and a relative increase in HDL-c are protective factors of the health of individuals with cardiometabolic disorders, even more so when experiencing stress.^1^Furthermore, a recent study shows that the high concentration of cortisol mediated the association between higher perceived stress and more severe MS, but only among individuals with poor coping skills.^1^ Some studies show an association between high cortisol levels and the prevalence of MS and abdominal fat, which was also found in this study. Likewise, a low ability to cope with stress was associated with an increased risk of developing DM2.^1^^,^^22^^,^^23^

Studies also indicate that an individual’s inability to deal with stress directly impacts the quality of life and health conditions at different stages of life, as well as favor the emergence of a cluster of risk factors for MS.^8^ The perception of chronic stress was significantly associated with MS criteria in Armborst et al.^2^ and another study shows that the stress perception of individuals with MS negatively impacts health-promoting behaviors.^7^^)^

Indeed, the role stress plays on an individual’s behavioral and physiological health condition is complex, and even more so in the presence of DM2 and MS. Blood pressure remains high in the presence of chronic stress, or more indirectly, due to an unhealthy lifestyle, with low exercise levels, a diet poor in nutrients, and alcohol consumption and smoking.^2^^,^^7^ These findings imply that a nurse-led health-promoting educational program focusing on MS adopting a multidisciplinary approach can promote decreased stress levels among adults with DM2 and MS within the PHC scope; stress levels significantly decreased among this study’s participants after the intervention. As reported in other studies,^2^^,^^6^^,^^9^ such an effect may be linked to the participants modifying their lifestyles.

Lifestyle changes involving a healthy diet and regular exercise are the primary approach in treating MS. Nonetheless, psychological stress should not be neglected, considering that coping strategies to deal with stress can be taught. Another potential explanation for these findings is that the intervention program was implemented in groups. In addition to encouraging a healthy lifestyle, group meetings also play an essential social role in a community.^6^ The group activities in our program seem to have positively influenced the participants’ health, particularly the MS parameters among middle-aged individuals. A clinically relevant finding is that highly stressed participants experienced positive effects on fasting blood glucose and HDL-c. The participants’ motivation in such programs improves the individuals’ adherence to the program and their ability to adapt to lifestyle changes, which promotes changes in the MS parameters. Note that the adults in this study presented a low health status, and their clinical conditions seemed to decrease their motivation to implement lifestyle changes.^6^^,^^9^

All participants were provided motivational counseling during the meetings and encouraged to make lifestyle changes to improve their MS parameters. Therefore, a multidisciplinary team of specialists with experience working with individuals with MS should keep this educational approach to health promotion in the long term. Furthermore, working with groups might improve clinical results by sensitizing patients regarding the interaction between stressors and cardiometabolic parameters and improving the patients’ ability to self-manage the disease.^6^ Naturally, as in any health-promoting educational approach, we cannot determine the extent to which individual project elements contributed to the outcome achieved in this study.

Long-term clinical studies addressing stressed*vs.*non-stressed individuals may provide a better understanding of variations in the syndrome parameters and indicate specific interventions to address early on cardiometabolic and mental health risks related to perceived stress. Therefore, current health policies aimed at people with DM2 and MS should focus on timely screening of perceived stress to improve therapeutic management within the PHC scope. Health units also need to expand strategies to deal with psychological stress, as proper coping strategies seem to play a relevant role in these individuals’ various health risks. Hence, our findings suggest that a health-promoting educational program can enable individuals with DM2 and MS to cope with stressful situations with greater psychological resilience. This finding is consistent with a previous study.^1^

This study’s sample size and short study period constitute limitations. For example, the small number of participants hindered an analysis of differences between sexes. For this reason, the generalization of results is limited and should be considered cautiously. Also, the few studies on the subject prevent comparisons. The predominance of women and their age leads us to note that the post-menopause period may cause mood changes and high stress levels, besides potential changes in cardiometabolic parameters, factors not assessed here.^6^ More extensive studies are expected to investigate perceived stress in different groups, verifying how different perceptions influence MS and mental health parameters in the long term.

The complexity of health problems involving MS emphasizes the need to develop effective strategies considering biopsychosocial aspects. The implementation of a health-promoting program in PHC units can be an effective educational mechanism to support the multidisciplinary treatment of MS among middle-aged adults with diabetes. Therefore, our results suggest that health policies and health workers assisting individuals with MS should consider stress perception in their practice and services rather than only focus on lifestyle to promote stress self-management strategies and improve coping and the MS parameters. Public policies should also consider developing health programs to monitor MS and help individuals improve their lifestyle, strengthening the instructions to maintain healthy behaviors and adapt to changes, harmoniously coexisting with their clinical condition. Finally, participation in health-promoting programs might be a support mechanism to deal with stressors, improving the individuals’ coping skills.

This study’s results directly affect nurses’ practice, highlighting the effectiveness of a nurse-led educational program to decrease perceived stress in adults with DM2 and MS. The active presence of nurses in PHC is crucial to promoting coping strategies, self-care, and lifestyle changes. The results also improve nurses’ daily practice, indicating the need for investing in these professionals’ training and performance. Thus, this study not only expands scientific knowledge in nursing but also provides a solid basis for the effective integration of educational programs led by nurses in primary health care to promote health and effectively manage chronic conditions such as DM2 and MS.

The conclusion is that the nurse-led health-promoting educational program to encourage lifestyle changes in the context of MS significantly decreased perceived stress among adults with diabetes and MS in a six-month intervention. Furthermore, the high perceived stress levels found in the participants in the intervention group seem to be associated with decreased blood glucose levels and increased HDL-c concentrations. Finally, the intervention group’s average number of MS criteria tended to decrease, regardless of the stress level. Nonetheless, long-term studies are needed to verify the differences in the groups’ perceived stress on the MS parameters and to investigate individual stress coping strategies.
